# Reagent-Free Colorimetric Cholesterol Test Strip Based on Self Color-Changing Property of Nanoceria

**DOI:** 10.3389/fchem.2020.00798

**Published:** 2020-09-02

**Authors:** Phuong Thy Nguyen, Young Im Kim, Moon Il Kim

**Affiliations:** Department of BioNano Technology, Gachon University, Seongnam-si, South Korea

**Keywords:** reagent-free colorimetric assay, paper strip, cholesterol determination, nanoceria, cholesterol oxidase

## Abstract

Paper-based test strip consisting of cerium oxide nanoparticles (nanoceria) as hydrogen peroxide (H_2_O_2_)-dependent color-changing nanozymes and cholesterol oxidase (ChOx) has been developed for convenient colorimetric determination of cholesterol without the need for chromogenic substrate. The construction of the cholesterol strip begins with physical adsorption of nanoceria on the paper surface, followed by covalent immobilization of ChOx via silanization, chitosan-mediated activation, and glutaraldehyde treatment of the nanoceria-embedded paper matrices. In the presence of cholesterol, ChOx catalyzes its oxidation to produce H_2_O_2_, which forms peroxide complex on the nanoceria surface and induces visual color change of the nanoceria-embedded paper from white/light yellow into intense yellow/orange, which was conveniently quantified with an image acquired by a conventional smartphone with the ImageJ software. Using this strategy, target cholesterol was specifically determined down to 40 μM with a dynamic linear concentration range of 0.1–1.5 mM under neutral pH condition, which is suitable to measure the serum cholesterol, with excellent stability during 20 days and reusability by recovering its original color-changing activity for 4 consecutive cycles. Furthermore, the practical utility of this strategy was successfully demonstrated by reliably determining cholesterol in human blood serum samples. This study demonstrates the potential of self color-changing nanozymes for developing colorimetric paper strip sensor, which is particularly useful in instrumentation-free point-of-care environments.

## Introduction

Cholesterol is a vital construction unit of animal cell membrane and key biosynthetic precursor of steroid hormone, bile acids, and vitamin D (Ikonen, 2008). It is also one of the most crucial biomarkers to diagnose various severe clinical disorders. A low level of cholesterol in human blood is closely related to hypolipoproteinemia, anemia, and septicemia, while elevated cholesterol level is linked to malnutrition hypertension, arteriosclerosis, brain thrombosis, and lipid metabolism dysfunction (Nauck et al., 1997; MacLachlan et al., 2000; Martin et al., 2003; Arya et al., 2008). Thus, it is crucially required to monitor cholesterol levels in clinical diagnosis for preventing these diseases. Until now, numerous analytical methods have been widely exploited for the sensitive quantification of cholesterol, based on high performance liquid chromatography, electrochemistry, fluorescence, chemiluminescence, and molecular imprinting polymer (MIP) technology (Lin et al., 2007; Zhang et al., 2008, 2012; Li et al., 2011; Matharu et al., 2012). However, these cholesterol detection methods require instrumentations with relatively complicated operating procedures, which limits their utilization in instrumentation-free environments. Hence, to facilitate routine cholesterol monitoring in point-of-care testing (POCT) environments, it is necessary to develop more simple strategy with enough sensitivity, reliability, and cost-effectiveness.

In this regard, colorimetric methods for cholesterol detection have gained considerable attention due to its visual detecting capability. Most of them have relied on natural peroxidase or peroxidase-like nanozyme, which combines with ChOx, and chromogenic substrate including 3,3′,5,5′-tetramethylbenzidine (TMB) or 2,2′-azino-bis(3-ethylbenzo-thiazoline-6-sulfonic acid) diammonium salt (ABTS) (Hayat et al., 2015; Kim et al., 2015; Wu et al., 2017; Chung et al., 2018). In the presence of cholesterol, ChOx oxidized cholesterol to produce H_2_O_2_, which subsequently activated peroxidase or peroxidase-mimicking material to oxidize TMB or ABTS into blue or green colored product, respectively. These methods enable facile detection with high selectivity and sensitivity; however, multiple components including the colorimetric reagents should be involved in the assay, and furthermore, spectrophotometer should be used for absorbance-based quantification of cholesterol.

Nanoceria have been also considered as one of the promising nanozymes for developing colorimetric assays since they catalyze the fast oxidation of colorimetric substrates to generate colored products even without the need for additional oxidizing agents (e.g., H_2_O_2_). Additionally, they have unique self-color transition property from white/light yellow into intense yellow/orange by H_2_O_2_-induced alteration of the oxidation state from Ce^3+^ to Ce^4+^ on the nanoparticle surface. Since the self-color change of nanoceria is induced only by H_2_O_2_ without any dye and the changed color of nanoceria can be returned to its original color due to the recovery of the ionic valence between Ce^3+^ and Ce^4+^, several colorimetric assays to detect H_2_O_2_ and other biomolecules including glucose and cholesterol with the aid of the corresponding oxidases have been reported (Ornatska et al., 2011; Kim et al., 2017). Although these examples demonstrate the potential of nanoceria as novel colorimetric transducers to detect H_2_O_2_ in the sample, they were still not free from spectrophotometer for quantification. Thus, to facilitate practical on-site cholesterol detection, we have developed a paper-based test strip which incorporates both nanoceria and ChOx for the determination of cholesterol level in human blood. Paper-based strip sensors have been widely exploited for colorimetric POCT applications owing to their unique advantages such as portability, disposability, low production cost, large surface area, easy surface functionalization, and strong background contrast for enhancing color signal intensity; however, until now, paper-based cholesterol strip has rarely been reported (Mahato et al., 2017). Various analytical characteristics of the test strip such as sensitivity, specificity, stability, reusability, and clinical utility in cholesterol detection were investigated with real images captured by a smartphone, which is quite suitable in POCT environments.

## Experimental Section

### Materials

Nanoceria (<5 nm in diameter), cholesterol oxidase from *Streptomyces* sp. (ChOx), cholesterol, glucose, uric acid, urea, cysteine, xanthine, galactose, triton X-100, sodium acetate, chitosan, succinic acid, aminopropyl triethoxysilane (APTES), and human blood serum were purchased from Sigma-Aldrich (Milwaukee, WI). Hydrogen peroxide (35 %) was obtained from Junsei Chemical Co. (Japan). Whatman grade 1 qualitative filter papers were purchased from GE Healthcare Co. (USA). All other chemicals were of analytical grade or higher and all solutions were prepared with distilled (DI) water purified using a Milli-Q Purification System (Millipore, USA).

### Preparation of Paper Strip Incorporating Nanoceria or Both Nanoceria and ChOx

Paper strip incorporating nanoceria or both nanoceria and ChOx was prepared according to previously reported procedures with slight modifications (Ornatska et al., 2011). First, nanoceria was fully dissolved in sodium acetate buffer (100 mM, pH 5.3) prior to the immobilization on test strip. Whatman filter paper was cut into rectangular strips, soaked in nanoceria solution (15 mg/mL) for 10 min, and dried for 3 h at 70°C. To stabilize nanoceria on the paper, the nanoceria-embedded strip was incubated in 5% APTES in ethanol for 10 min and dried for 10 min at 100°C, which was then used for colorimetric H_2_O_2_ detection.

For additional ChOx immobilization on the paper, the silanized nanoceria-embedded paper was soaked in chitosan solution (1% chitosan in 0.5% aqueous succinic acid solution) for 10 min and allowed to dry for 10 min at room temperature (RT). Then, the paper was treated with 5% aqueous glutaraldehyde solution for 20 min, washed five times with DI water, and dried for 10 min at RT. Finally, the paper strip was soaked into ChOx solution (6 mg/mL in sodium acetate buffer (100 mM, pH 5.3). After 20 min, the paper was washed with PBS buffer (10 mM, pH 7.4) three times, dried at RT for 10 min, and then used directly or stored at 4°C for further experiments.

The shape and particle size of the nanoceria was checked by transmission electron microscopy (TEM) analysis. For the TEM, 5 μL of nanoceria solution was applied by drop casting the particle suspensions on a carbon-coated copper TEM grid (Electron Microscopy Sciences, USA) followed by drying at RT. The prepared sample was observed using field emission TEM (Tecnai, FEI) with accelerating voltages up to 200 kV. The morphologies and elemental distributions of the paper strip incorporating both nanoceria and ChOx were analyzed by scanning electron microscopy (SEM, Magellan 400) and energy dispersive X-ray spectroscopy (EDX) imaging modes. For SEM analysis, the paper strips were freeze-dried for 2 days and analyzed on SEM. X-ray photoelectron spectroscopy (XPS) (Thermo Scientific, WI) was performed to examine the surface chemical properties of nanoceria. Atomic force microscopy (AFM) was also conducted in the contact mode with a bio atomic force microscope (JPK NanoWizard II, Germany) to characterize the surface morphology of paper strips.

### Colorimetric Detection of H_2_O_2_ and Cholesterol Using Nanoceria-Embedded Paper Strip

H_2_O_2_ level was quantified by soaking the nanoceria-embedded paper strip into aqueous sample solutions containing various concentrations of H_2_O_2_ for 3 min at RT. The images of resultant strip were then acquired using a smartphone (GALAXY S8 NOTE, Samsung), followed by converting to cyan-magenta-yellow-black (CMYK) mode, which was subjected to quantitative image processing with the ImageJ software (NIH). For cholesterol determination, cholesterol stock was first prepared by dissolving it in the mixture of isopropanol and Triton X-100 (1:1, v/v), and subsequently diluted with buffer (PBS, pH 7.4) to obtain cholesterol solutions. The paper strip incorporating both nanoceria and ChOx was soaked into the sample solutions containing various concentrations of cholesterol for 50 min at RT. The reacted paper strip was directly used to obtain images with a smartphone and the other procedures were the same as those described for H_2_O_2_ determination.

Long-term storage stability of the cholesterol sensing strips was assessed by incubating them at different conditions (RT, 4°C, and −20°C), and their residual activities were determined at predetermined time points by measuring their color intensities toward 10 mM cholesterol as described above. Reusability was also evaluated after cycles involving the typical reaction with 10 mM cholesterol and twice washings with aqueous buffer (100 mM sodium acetate, pH 5.3) to remove unreacted cholesterol on the paper. After recovering initial color of the paper strips by incubating them at RT for 5 days, the strip was reused for the measurement of residual color-changing activity toward cholesterol. The relative color intensity (%) was calculated based on the ratio of the residual color intensity to the original one.

For the determination of the cholesterol level in human serum, the original amount of cholesterol in serum was first determined using cholesterol assay kit (Sigma-Aldrich). Predetermined amount of cholesterol was further added into serum to make spiked samples representing normal, boundary, and high levels of cholesterol in blood serum. The concentration of cholesterol in each spiked sample (5-fold dilution) was measured using the same procedures as described above. The recovery rate [recovery (%) = measured value/actual value × 100] and the coefficient of variation [CV (%) = SD/average × 100] were assessed to determination the precision and reproducibility of the paper strip assay.

## Results and Discussion

### Construction of Reagent-Free Cholesterol Sensing Paper Strip

**Figure 1:**
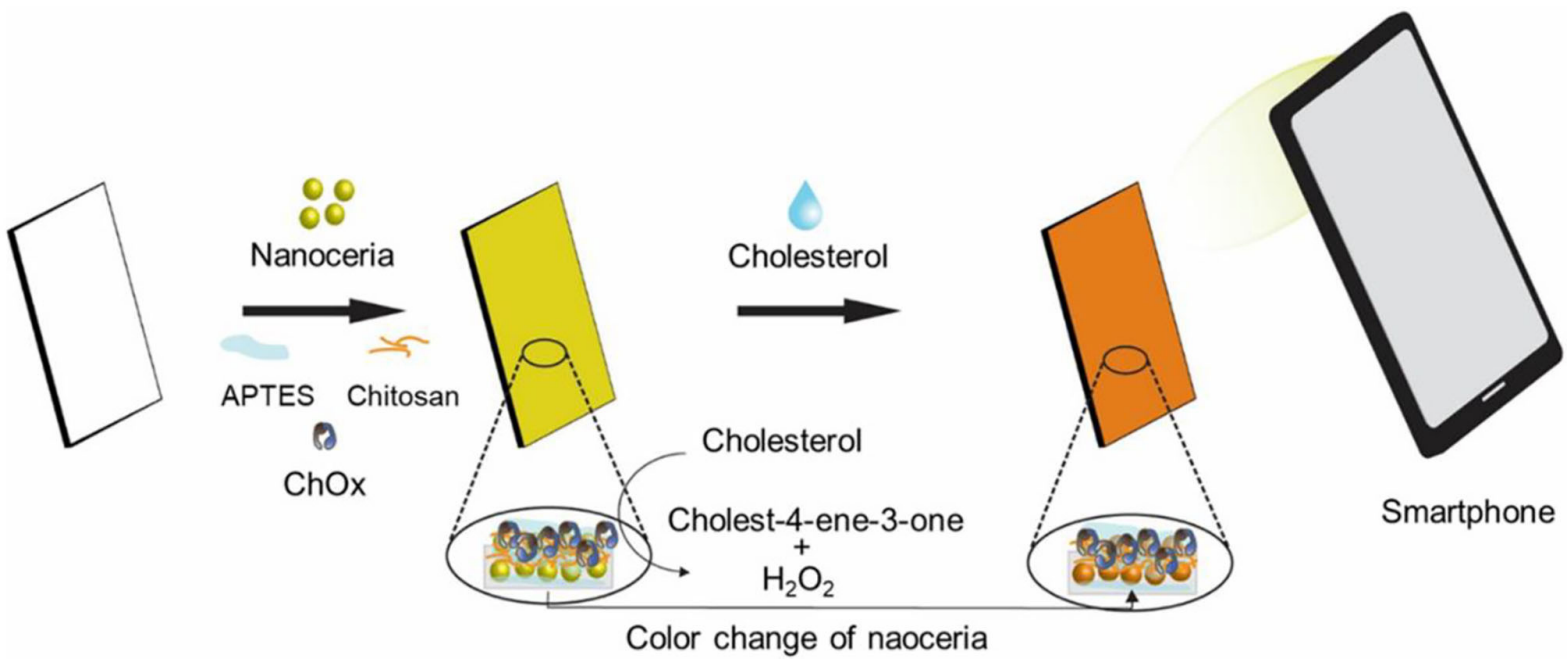
Schematic illustration of the paper strip incorporating nanoceria and ChOx for the smartphone-mediated reagent-free colorimetric determination of cholesterol.

A cholesterol sensing paper strip incorporating both nanoceria and ChOx was developed for convenient reagent-free identification of cholesterol in human blood serum. It was constructed by physically immobilizing nanoceria on the paper matrices, followed by APTES-mediated stabilization, chitosan and glutaraldehyde-mediated activation, and covalent immobilization of ChOx. APTES would form siloxane bridges with the hydroxyl groups of cellulose fibers of the paper matrices, which facilitates hydrogen bonding with the hydroxylated nanoceria surfaces, consequently yielding strong attachment of nanoceria on the paper matrices. Chitosan and glutaraldehyde treatments would also provide rich amine moieties and active aldehyde groups, respectively, both of which efficiently induce covalent linkages with amine groups of ChOx. We envisioned that the paper strip incorporating both nanoceria and ChOx would serve as an efficient colorimetric cholesterol biosensor capable of being used for determining cholesterol level in human blood. In the presence of cholesterol, ChOx on the strip catalyzes the oxidation of cholesterol to produce H_2_O_2_, which reacts with nanoceria and induces their vivid color change from white/light yellow into intense yellow/orange without any addition of chromogenic substrate. This color change is owing to the H_2_O_2_-mediated transition of the oxidation state of nanoceria from Ce^3+^ to Ce^4+^ and the formation of peroxide complex at the nanoceria surface (Scholes et al., 2006; Karakoti et al., 2009; Singh et al., 2011). Through the XPS analysis, we observed a significant decrease in the Ce^3+^/Ce^4+^ ratio of nanoceria after the addition of H_2_O_2_ (Figure S1), that clearly demonstrates the above color transition mechanism. The images of resulting paper strip were acquired using a smartphone, and quantitative information was obtained by simple image processing with ImageJ software (Figure 1).

**Figure 2:**
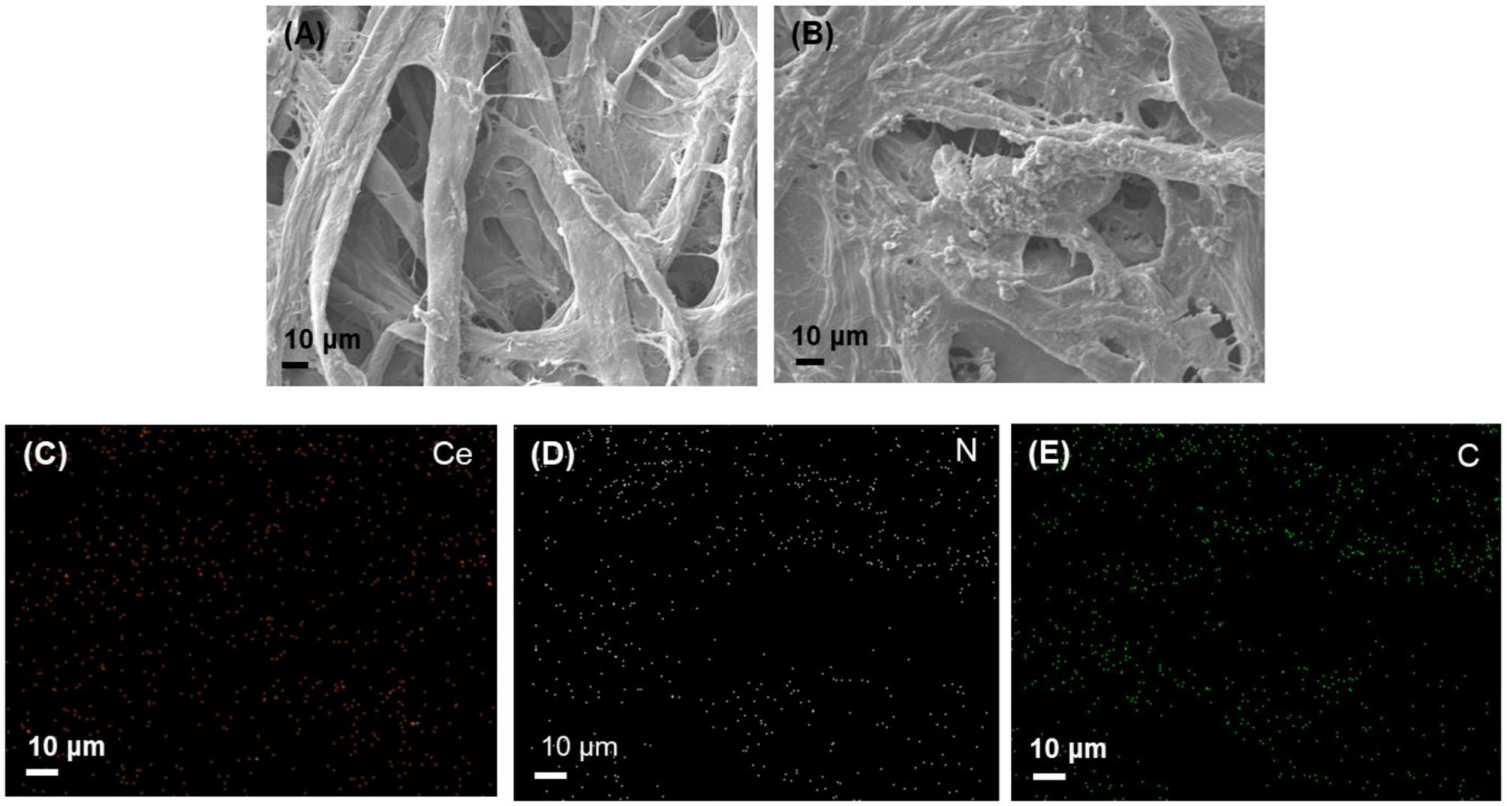
SEM images of **(A)** bare paper strip, **(B)** paper strip incorporating both nanoceria and ChOx, and the corresponding EDS maps of **(C)** Ce, **(D)** N, and **(E)** C elements.

The morphologies and elemental distributions were analyzed by TEM and SEM with EDX (Figure S2 and Figure 2). TEM image showed the spherical shape of the nanoceria with a size of ~10 nm. From the SEM images, we could see much more irregular surfaces having particle-like dots in the paper strip incorporating both nanoceria and ChOx, while relatively smooth surfaces were observed from the bare paper strip, indicating the effective loading of nanoceria and ChOx on the paper strip. Elemental mapping images of Ce, N, and C elements also demonstrated that nanoceria and ChOx were homogeneously distributed throughout the paper matrices, which may contribute to an enhanced colorimetric response compared with aggregated cases.

### Analytical Capability of Paper Strip for the Determination of H_2_O_2_ and Cholesterol

**Figure 3:**
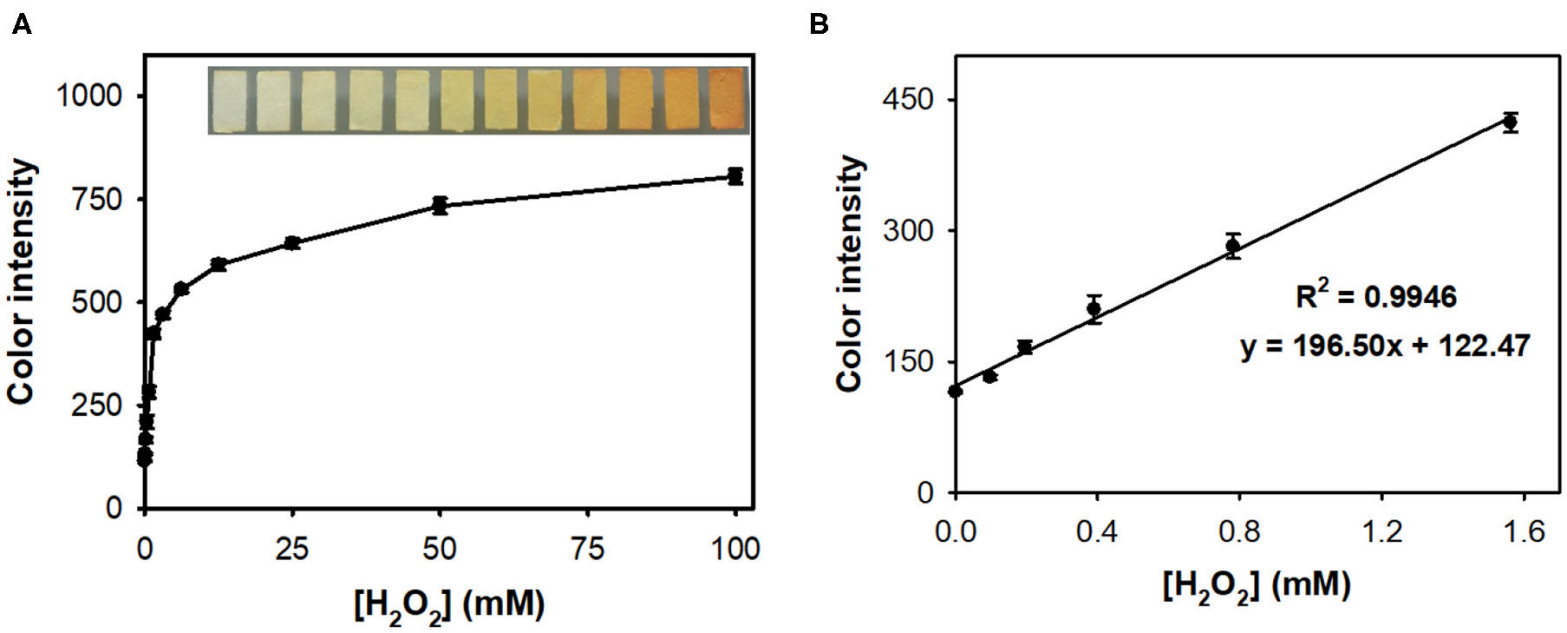
**(A)** Real images and the corresponding dose-response curve for H_2_O_2_ determination using nanoceria-embedded paper strip and **(B)** linear calibration plot. The error bars represent the standard deviation of three independent measurements.

The colorimetric responses of nanoceria-embedded paper strip toward H_2_O_2_ were first assessed by soaking the paper strip into sample solutions containing diverse levels of H_2_O_2_, followed by acquiring images using smartphone (Figure 3). ImageJ software was utilized to convert the real images of paper strips into the CMYK mode for quantifying their color intensity. By simple 3 min reaction at RT, the paper strip showed clear color change from white to intense yellow, proportional to the H_2_O_2_ levels (Figure 3A). The color intensity increased with increasing H_2_O_2_ concentration in the sample, with a dynamic linear range of 0.1–1.5 mM (*R*^2^ = 0.9946), and the limit of detection (LOD) was calculated to be as low as 0.05 mM (Figure 3B), which is enough for coupling with ChOx to create cholesterol assay system (Kim et al., 2011).

**Figure 4:**
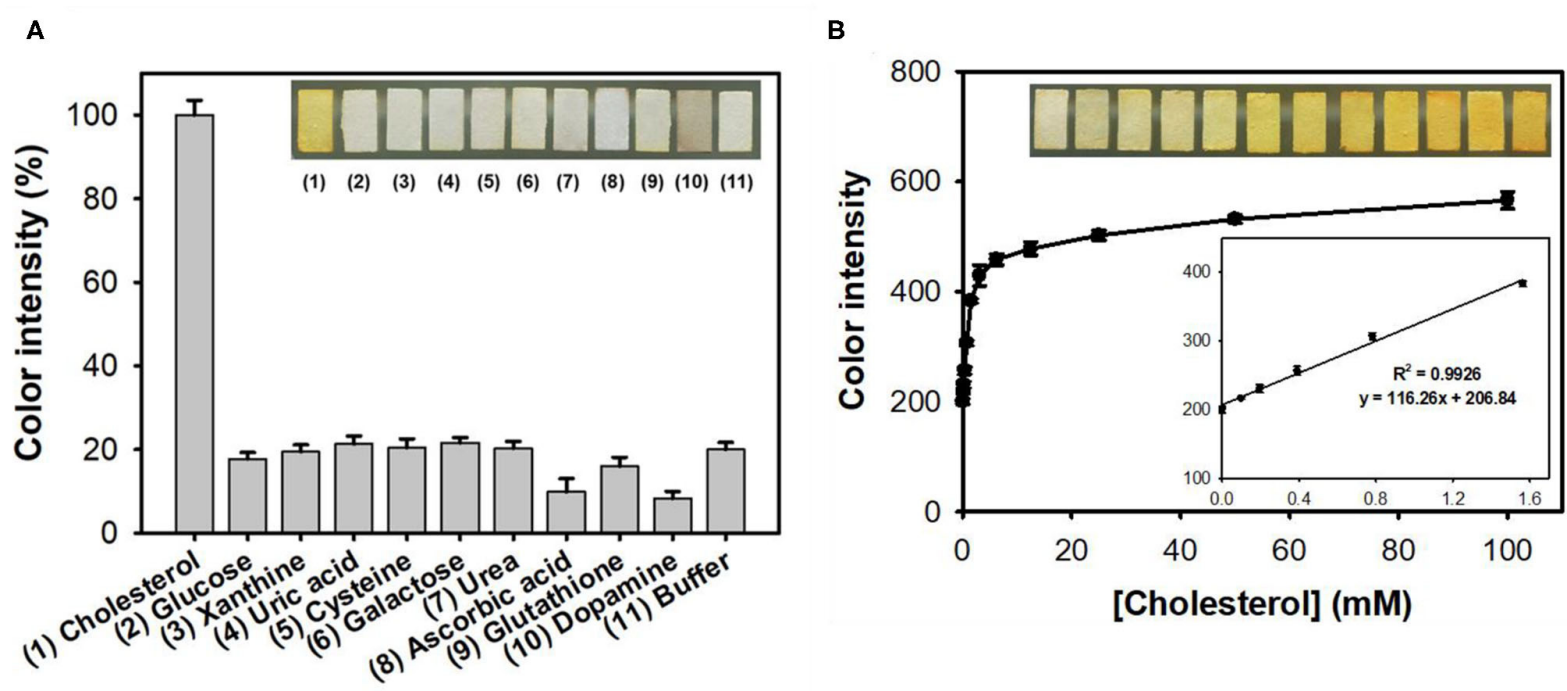
**(A)** Real images and the corresponding color intensities of the selective colorimetric detection of cholesterol using paper strips incorporating nanoceria and ChOx. A 10 mM concentration of cholesterol was used, while 100 mM of other substrates was used in the experiments. **(B)** Real images, dose-response curve, and the corresponding linear calibration plots for cholesterol determination using paper strips incorporating nanoceria and ChOx. The error bars represent the standard deviation of three independent measurements.

The feasibility of the paper strip incorporating both nanoceria and ChOx was then demonstrated, for colorimetric determination of target cholesterol without involvement of any chromogenic substrate. Although the nanoceria-embedded paper strip incubated with free ChOx without immobilization yielded slightly higher level of color transition toward cholesterol, it is not convenient to use since ChOx was not immobilized on the paper matrices (Figure S3). Investigations for the effects of experimental parameters such as temperature, buffer pH, and incubation time on the color intensity of paper strip toward cholesterol showed that 37°C, pH 7, and incubation for 50 min were the ideal assay conditions (Figure S4). Although the incubation at 37°C yielded the maximal color intensity, we incubated our paper strip with cholesterol at RT rather than 37°C because of the practical convenience and sufficiently high color intensity at RT (over 90% compared to that from 37°C). Through the simple soaking of the paper strip into the sample solutions, cholesterol was specifically detected by generating intense yellowish color, while no significant color change was observed from the common interfering substances such as glucose, xanthine, uric acid, cysteine, galactose, urea, ascorbic acid, glutathione, and dopamine, which commonly appear in human blood, even at 10-fold higher concentrations to that of cholesterol (Figure 4A). Moreover, these interfering compounds did not hinder the color change of the paper strips even when they were co-presented with cholesterol (Figure S5). In case of dopamine, although there was a different dark color observed from the paper strip, we could obtain similar yellowish color intensity using the ImageJ software.

Through the analysis of dose-response curve, the LOD for cholesterol was determined to be 0.04 mM, with a dynamic linear range from 0.1 to 1.5 mM (Figure 4B). The LOD value of the assay system was calculated based on the formula: LOD = 3 × δ/slope, where δ is the standard deviation of blank and slope is the slope of calibration curve (Kim et al., 2018). The LOD and linear range values of our paper strips are among the best describing colorimetric detection of cholesterol (Table S1). Furthermore, our system solely enables reagent-free colorimetric determination of cholesterol, which is quite advantageous in practical applications. Considering that the cut-off value of hypercholesterolemia patients is about 6 mM, thus the current cholesterol paper strip is suitable to distinguish patients with hypercholesterolemia and normal persons (Nair et al., 2014). Although the employed nanoceria and ChOx were not uniformly immobilized but aggregated to some extent on paper matrices (Figure S6), the detection performances of the developed cholesterol paper strip were enough for practical applications.

**Figure 5:**
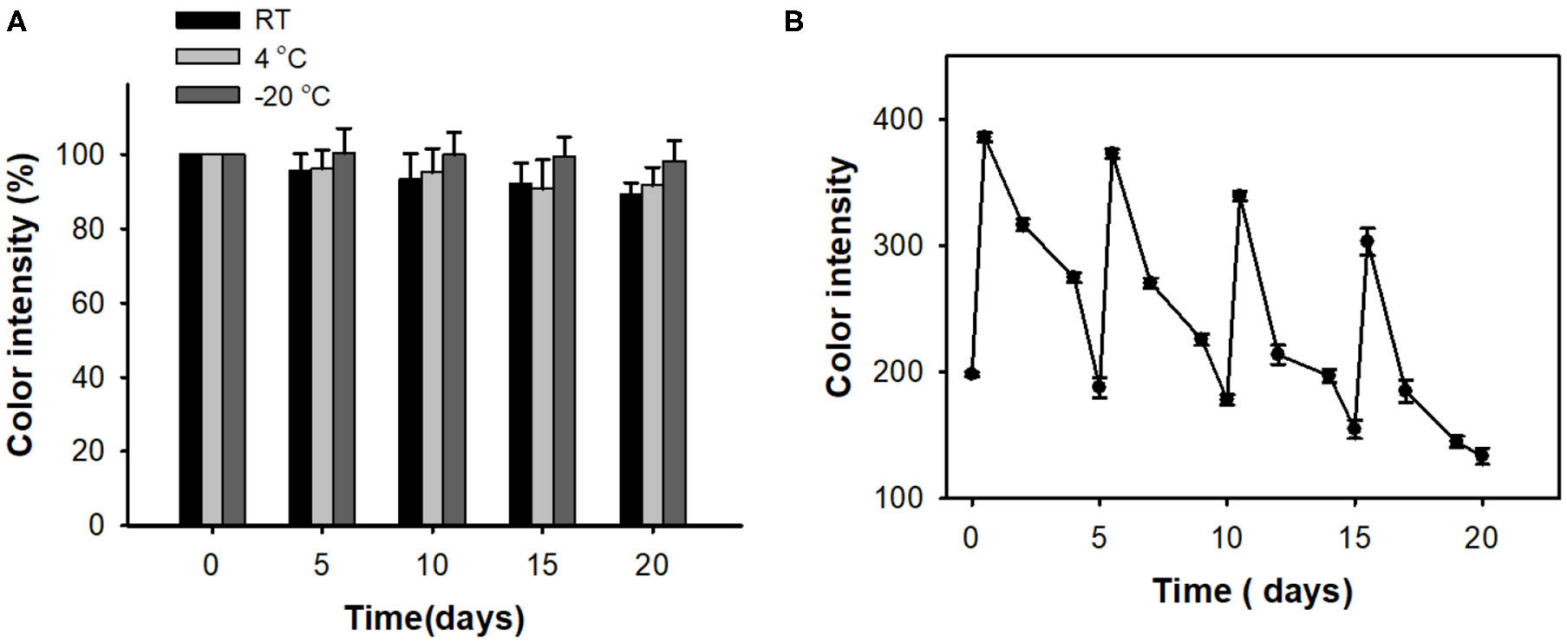
**(A)** Long-term storage stability and **(B)** reusability of paper strips for cholesterol detection.

We also evaluated long-term storage stability and reusability of the developed cholesterol paper strip. The storage stabilities were examined by measuring the residual color intensities toward cholesterol, during the storages in three conditions at RT, 4°C, and −20°C. The investigations clearly indicated that the paper strip showed excellent storage stability during 20 days (Figure 5A). When incubating at RT, our paper strip showed slight decrease in color intensity; however, over 90% of initial activity still remained at 20 days of incubation. The paper strip is also expected to be reused for multiple times owing to the expected decomposition of the adsorbed peroxide species on the surface of embedded nanoceria (Kim et al., 2017). As a result, the paper strip fully regained its original color up to 4 consecutive cycles (Figure 5B). However, its maximal color intensity after reaction was gradually decreased, indicating that the immobilized state of nanoceria on the paper matrices would hinder the reversibility of nanoceria.

### Determination of Cholesterol Levels in Human Serum Samples

Finally, we examined the diagnostic capability of the paper strip incorporating nanoceria and ChOx, using clinical human serum samples containing representative levels of cholesterol (normal; ≤ 5 mM, boundary; 5–6 mM, and high; >6 mM) (Nair et al., 2014). The original amount of cholesterol in the serum samples was first determined using a cholesterol assay kit, and a predetermined amount of cholesterol was added to establish the representative levels. 10-fold dilution was applied to the prepared samples to adjust their cholesterol concentration within our linear range. According to the experimental results, the serum cholesterol levels were quantitatively determined with excellent precision, yielding CVs ranging from 3.97 to 5.67% and recoveries ranging from 97.39 to 101.19% (Table 1), validating the excellent reproducibility and reliability of this assay. The precision values were similar to those obtained with commercially-available cholesterol assay kit (Table S2). These results demonstrate that the proposed reagent-free colorimetric cholesterol paper strip can be employed as a promising analytical tool for convenient identification and first screening of hypercholesterolemia in POCT environments.

**Table 1:** Detection precision of the paper strip incorporating nanoceria and ChOx for the quantification of cholesterol levels in spiked human serum samples.

	**Original amount (mM)**	**Added (mM)**	**Expected (mM)**	**Measured^**a**^ (mM)**	**SD^**b**^**	**CV^**c**^ (%)**	**Recovery^**d**^ (%)**
**Normal**		1	2.08	2.08	0.08	3.97	99.84
**Boundary**	1.08	4.5	5.58	5.43	0.31	5.67	97.39
**High**		6	7.08	7.16	0.33	4.58	101.19

a *The average value of 5 successive measurement experiments*.

b *standard deviation (SD) of 5 measurements*.

c *Coefficient of variation = (SD/mean) × 100*.

d *Recovery = (Measured value/Expected value) × 100*.

## Conclusions

We herein developed a paper strip sensor incorporating both nanoceria and ChOx, for reagent-free as well as instrumentation-free determination of cholesterol. The paper strip displayed excellent selectivity, sensitivity, and linearity for the determination of target cholesterol by simple processing the real images acquired using a smartphone, with excellent storage stability and reusability. The clinical utility of the strip sensor was successfully demonstrated by reliably determining the cholesterol levels from clinical human serum samples. Since the current nanoceria-embedded paper strip enabled visual detection of the target cholesterol without involvement of any chromogenic dye or detection instrumentation, it should find practical applications in POCT environments.

## Data Availability Statement

The original contributions presented in the study are included in the article/Supplementary Material, further inquiries can be directed to the corresponding author/s.

## Author Contributions

PN and YK performed investigation. PN wrote the original draft. MK performed conceptualization, supervision, and reviewing the manuscript. All authors contributed to the article and approved the submitted version.

## Conflict of Interest

The authors declare that the research was conducted in the absence of any commercial or financial relationships that could be construed as a potential conflict of interest.
