# Supplementary material for: Reagent-Free Colorimetric Cholesterol Test Strip Based on Self Color-Changing Property of Nanoceria
Source: Front Chem. 2020 Sep 2;8:798. doi: 10.3389/fchem.2020.00798 (PMC7493407; doi:10.3389/fchem.2020.00798)
Supplement: Supplementary file 1 [file Data_Sheet_1.docx]

Supplementary Material

Supplementary Figure 1:


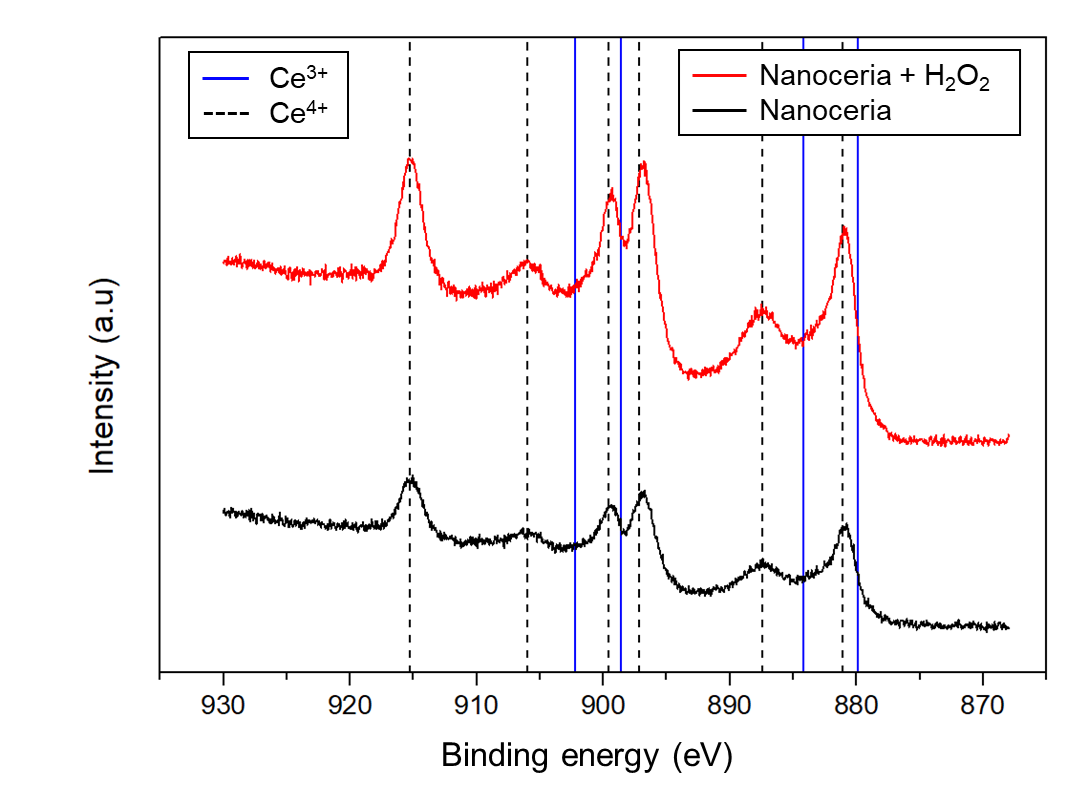


**Figure S1.** XPS spectra of Ce 3d of nanoceria and nanoceria after reaction with H_2_O_2_ (100 mM).


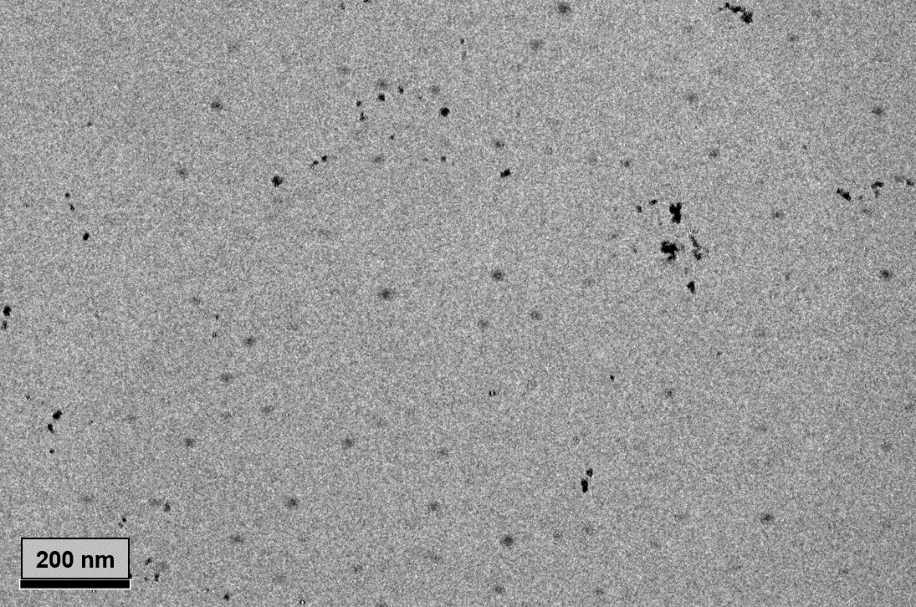


**Figure S2.** TEM image of free nanoceria.


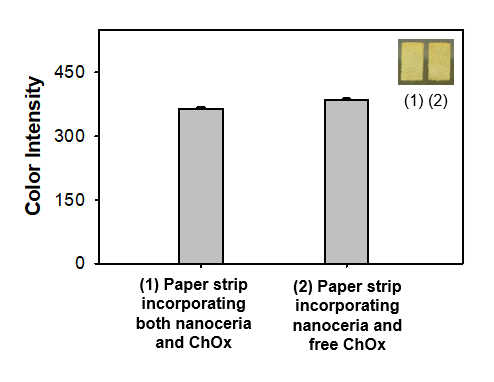


**Figure S3.** Real images and the corresponding color intensities of cholesterol (10 mM) detection using (1) paper strip incorporating both nanoceria and ChOx and (2) paper strip incorporating nanoceria, which is just incubated with free ChOx.


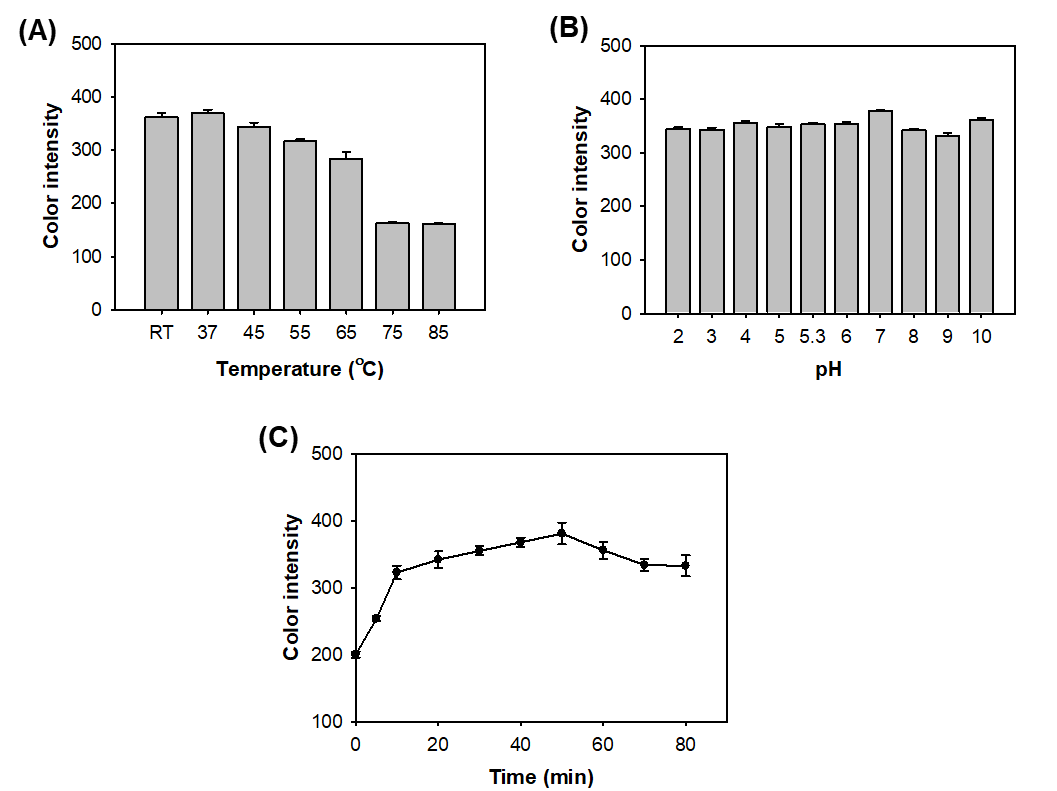


**Figure S4.** Effects of **(A)** temperature, **(B)** pH and **(C)** incubation time on the level of color intensity for cholesterol detection.


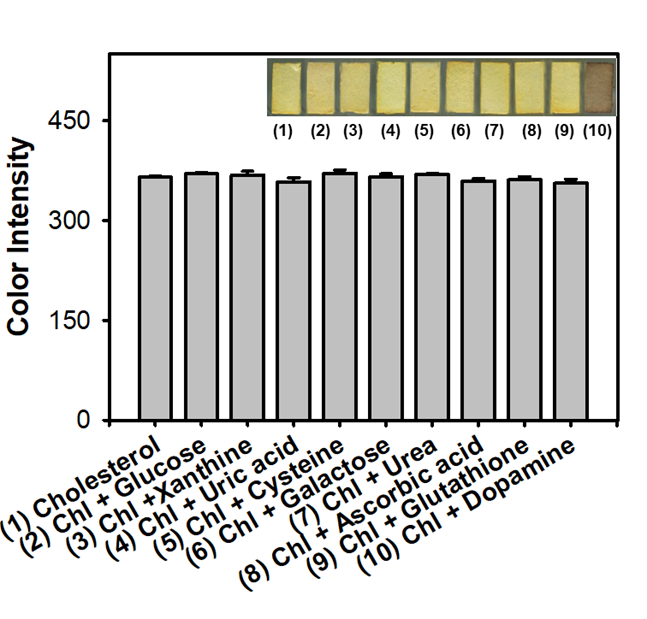


**Figure S5.** Real images and the corresponding color intensities of the selective colorimetric detection of cholesterol in the co-existence of interfering compounds, using paper strips incorporating nanoceria and ChOx. A 10 mM concentration of cholesterol (Chl) and 100 mM of interfering compounds were used in the experiments.


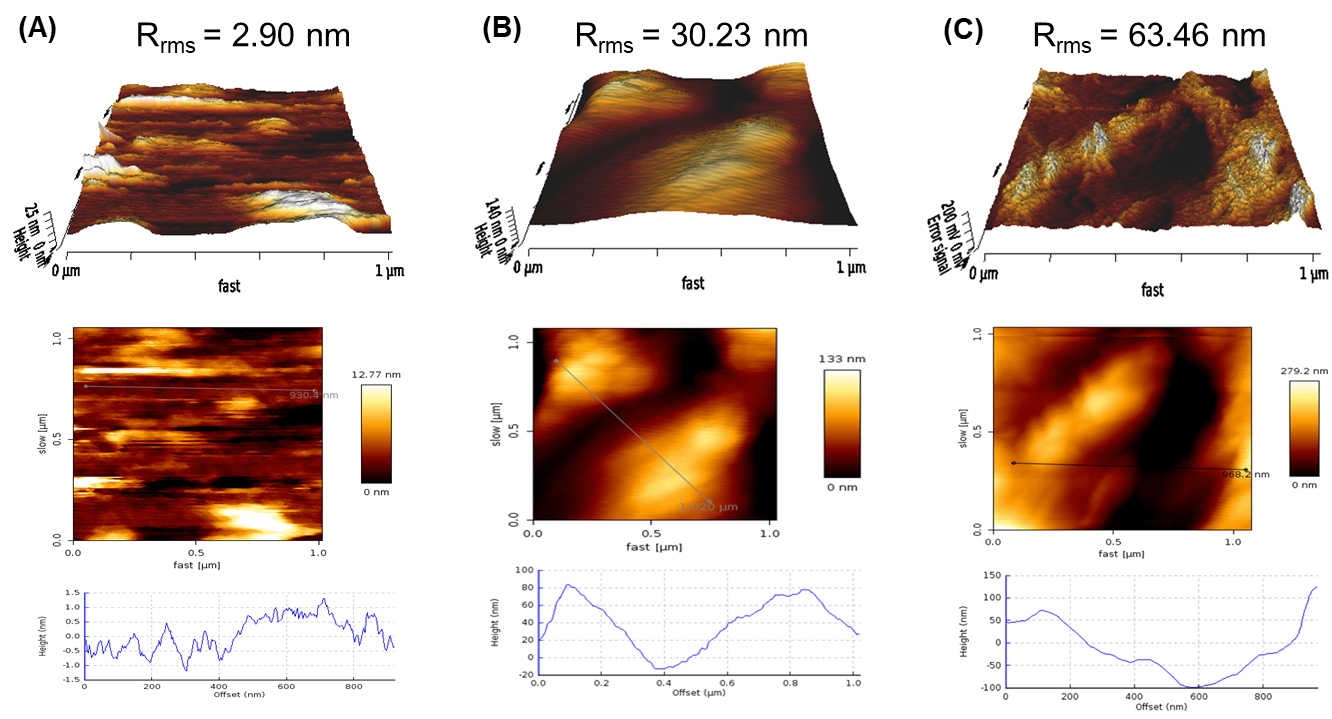


**Figure S6.** 3D topographical AFM images and the corresponding root-mean-square roughness (R_rms_) of **(A)** bare paper strip, **(B)** paper strip incorporating nanoceria, and **(C)** paper strip incorporating both nanoceria and ChOx.

**Table S1.** An overview on the LOD and linear range values of colorimetric determination of cholesterol.

| **Sensing materials** | **Linear range (mM)** | **LOD (mM)** | **References** |
| --- | --- | --- | --- |
| ChOx/Magnetic mesoporous silica | 0.39-6.58 | 0.19 | Kim et al. 2015 |
| ChOx/Au@Ag NPs | 0.3-300 | 0.15 | Zhang et al. 2016 |
| CuO/Graphene nanosphere | 0.1-0.8 | 0.078 | Sharma et al. 2017 |
| Chitosan nanofibers/ChOx | 1.29-7.75 | 1.29 | Dhawane et al. 2019 |
| Pc(OH)_8_/CoSn(OH)_6_ | 0.4-0.8 | 0.0914 | Lyu et al. 2020 |
| Paper strips incorporating nanoceria and ChOx | 0.1-1.5 | 0.04 | This work |

**Table S2.** Detection precision of the commercially-available cholesterol assay kit (Sigma-Aldrich) for the quantification of cholesterol levels in spiked human serum samples.

|  | **Original**  **amount**  **(mM)** | **Added**  **(mM)** | **Expected**  **(mM)** | **Measured^a^**  **(mM)** | **SD^b^** | **CV^c^**  **(%)** | **Recovery^d^**  **(%)** |
| --- | --- | --- | --- | --- | --- | --- | --- |
| **Normal** | 1.08 | 1 | 2.08 | 2.07 | 0.11 | 5.35 | 99.29 |
| **Boundary** |  | 4.5 | 5.58 | 5.49 | 0.40 | 7.37 | 98.47 |
| **High** |  | 6 | 7.08 | 6.93 | 0.12 | 1.69 | 97.90 |

^a^ The average value of 5 successive measurement experiments. ^b^ standard deviation (SD) of 5 measurements. ^c^ Coefficient of variation = ( SD / mean ) $\times$ 100. ^d^ Recovery = ( Measured value / Expected value ) $\times$ 100

**References**

Dhawane, M., Deshpande, A., Jain, R. and Dandekar, P. (2019). Colorimetric point-of-care detection of cholesterol using chitosan nanofibers. Sens Actuators B Chem. 281. doi: 10.1016/j.snb.2018.10.060

Lyu, H., Yin, D., Zhu, B., Lu, G., Liu, Q.Y., Zhang, X. and Zhang, X. (2020). Metal-free 2(3),9(10),16(17),23(24)-octamethoxyphthalocyanine modified uniform CoSn(OH)_6_ nanocubes: enhanced peroxidase-like activity, catalytic mechanism and fast colorimetric sensing for cholesterol. ACS Sustain. Chem. Eng. 8:25. doi: 10.1021/acssuschemeng.0c02151

Kim, M.I., Cho, D., and Park, H.G. 2015. Colorimetric quantification of glucose and cholesterol in human blood using a nanocomposite entrapping magnetic nanoparticles and oxidases. J. Nanosci. Nanotechnol. 15:10. doi: 10.1166/jnn.2015.11227

Sharma, V. and Mobin, S.M. (2017). Cytocompatible peroxidase mimic CuO: graphene nanosphere composite as colorimetric dual sensor for hydrogen peroxide and cholesterol with its logic gate implementation. Sens Actuators B Chem. 240. doi: 10.1016/j.snb.2016.08.169

Zhang, X., Wei, M., Lv, B., Liu, Y., Liu, X. and Wei, W. (2016). Sensitive colorimetric detection of glucose and cholesterol by using Au@ Ag core–shell nanoparticles. RSC Adv. 6:41. doi: 10.1039/C6RA04976A
